# High ionic conduction, toughness and self-healing poly(ionic liquid)-based electrolytes enabled by synergy between flexible units and counteranions†

**DOI:** 10.1039/d1ra04553a

**Published:** 2021-11-03

**Authors:** Fu Jie Yang, Qing Feng Liu, Xiao Bing Wu, Yu Yi He, Xu Gang Shu, Jin Huang

**Affiliations:** College Chemistry and Chemical Engineering, Zhongkai University of Agriculture and Engineering Guangzhou 510275 P. R. China yangfujie580@163.com; College of Pharmacy, Guangxi Medical University Nanning 530021 P. R. China huangjin@mailbox.gxnu.edu.cn

## Abstract

Polymer electrolytes offer great potential for emerging wearable electronics. However, the development of a polymer electrolyte that has high ionic conductivity, stretchability and security simultaneously is still a considerable challenge. Herein, we reported an effective approach for fabricating high-performance poly(ionic liquids) (PILs) copolymer (denoted as PIL-BA) electrolytes by the interaction between flexible units (butyl acrylate) and counteranions. The introduction of butyl acrylate units and bis(trifluoromethane-sulfonyl)imide (TFSI^−^) counteranions can significantly enhance the mobility of polymer chains, resulting in the effective improvement of ion transport, toughness and self-healability. As a result, the PIL-BA copolymer-based electrolytes containing TFSI^−^ counterions achieved the highest ionic conductivity of 2.71 ± 0.17 mS cm^−1^, 1129% of that of a PIL homopolymer electrolyte containing Cl^−^ counterions. Moreover, the PIL-BA copolymer-based electrolytes also exhibit ultrahigh tensile strain of 1762% and good self-healable capability. Such multifunctional polymer electrolytes can potentially be applied for safe and stable wearable electronics.

## Abstract 2

Polymer electrolytes offer great potential for emerging wearable electronics.

## Graphical abstract

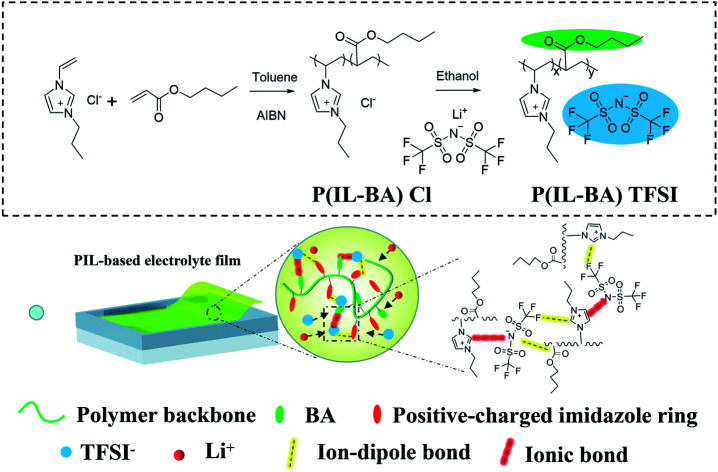

## Introduction

With the rapid development of wearable electronic devices, flexible solid-state batteries possessing high energy density have captured both academic and industrial attention in recent years.^1,2^ As one of the key components in flexible batteries, solid polymer electrolytes (SPEs) are inevitably subjected to external deformations like stretching, folding and bending in practical use, which may easily lead to damage of the SPEs that hinders the transportation of ions between the cathode and the anode, and severely deteriorates the battery performance.^3,4^ Assuming that the SPEs can be endowed with self-healing function, it will automatically repair cracks and damage. This will largely improve the reliability of flexible solid-state batteries.

Based on the above motivation, quite a few self-healable SPEs containing the covalent or non-covalent bonds such as disulfide bonds,^5,6^ hydrogen bonds,^7–9^ and ionic bonds,^10–12^ have been identified in the past decades. Among several different classes, polymerized ionic liquids or so-called poly(ionic liquid)s (PILs) formed from ionic liquid (IL) monomers are a promising candidate for intrinsic self-healing SPEs with the reversible ionic interactions between the charged polymer backbones and counter-ions.^13–15^ Moreover, PILs have been applied in some electrochemical devices like sensors, dye-sensitized solar cells, and lithium ion batteries, because they combine the mechanical durability of the polymer with the electrochemical properties of the ionic liquids.^16–19^ However, the ionic conductivities of PILs are relatively lower than that of the corresponding ILs,^20–22^ leading to the deterioration of the whole performance of assembled devices. In the past few years, it was found that in PILs, ion transport and self-repairing capability are largely dependent on the mobility of polymer chains.^8,23^ Various efficient measures like plasticizing,^24^ interpenetrating,^25^ and copolymerizing^26^ were utilized to decrease the glass-transition temperature (*T*_g_) of polymers for promoting the movement of polymer chains. Among them, in the reported strategy of copolymerization, the polymer backbones of SPEs were chemically modified by the effective functional groups on the molecular level. In this way, the SPEs were obtained with high ionic conductivity, good electrochemical stability and excellent mechanical performance while improving the mobility of polymer chains.^27^ It has been reported that the *T*_g_ values of polymers can be significantly decreased by grafting the ester groups into the main chains of the polymer through copolymerization.^28–30^ For instance, Guo *et al.* reported PIL copolymers containing ethyl acrylate units with excellent healing properties at 55 °C due to the higher mobility of the counteranions and the polymer chains.^30^ However, the prepared PIL copolymer as polymer electrolyte shows an low ionic conductivity (1.6 × 10^−7^ S cm^−1^) at 25 °C, which is far from the require of batteries. Most recently, Cai and coworkers have prepared a PIL-based quasi-solid state copolymer electrolyte for lithium–sulfur batteries by polymerization of butyl acrylate (BA) with IL monomer and crosslinked with poly(ethylene glycol)diacrylate.^31^ The main objective of such copolymer electrolyte system is to use abundant ester groups of the BA component to capture lithium polysulfides for improving the initial discharge capacity, cycle stability and rate performance of the assembled battery. However, the PIL-based copolymer chains may exhibit deficient movability due to covalently crosslinked networks. In addition, the self-healing properties and the contribution of ester groups to ion conduction were not discussed on the above copolymer electrolyte system.

**Scheme 1:**
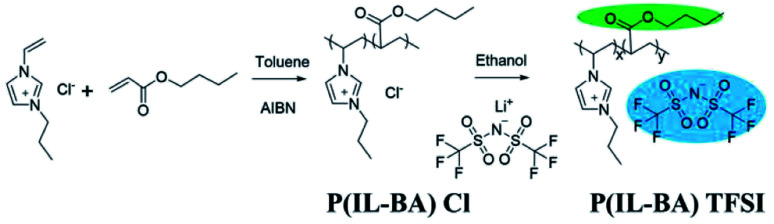
Schematic illustration of the synthesis of the PIL-BA copolymers containing different counter-ions (Cl^−^ or TFSI^−^).

In this work, a novel PIL-based copolymer electrolyte containing ester groups by copolymerizing imidazolium monomers with butyl acrylate (BA) is prepared. The overall fabrication process is schematically illustrated in Scheme 1. Considering the interaction between main chains of polymer and counter-ions in the PIL-based system,^32^ the electrolytes with different counter-anions such as Cl^−^ and TFSI^−^ are also synthesized by an ion-exchange reaction at the same time. The PIL-BA electrolyte membranes with different counteranions (Fig. S1†) can be easily fabricated by using a simple solvent-casting method using the ethanol solutions of prepared PIL-BA copolymers (Fig. S2†). Combining different counteranions and BA will improve the movement of the polymer chains. As a result, compared with PIL homopolymer electrolytes, the PIL-based copolymer electrolytes exhibit high ionic conductivity, excellent mechanical properties and good self-healable capability. It is hoped that the as-prepared PIL copolymer electrolytes could offer new insight into the development of the advanced high-safe polymer electrolytes.

## Results and discussion

**Fig. 1:**
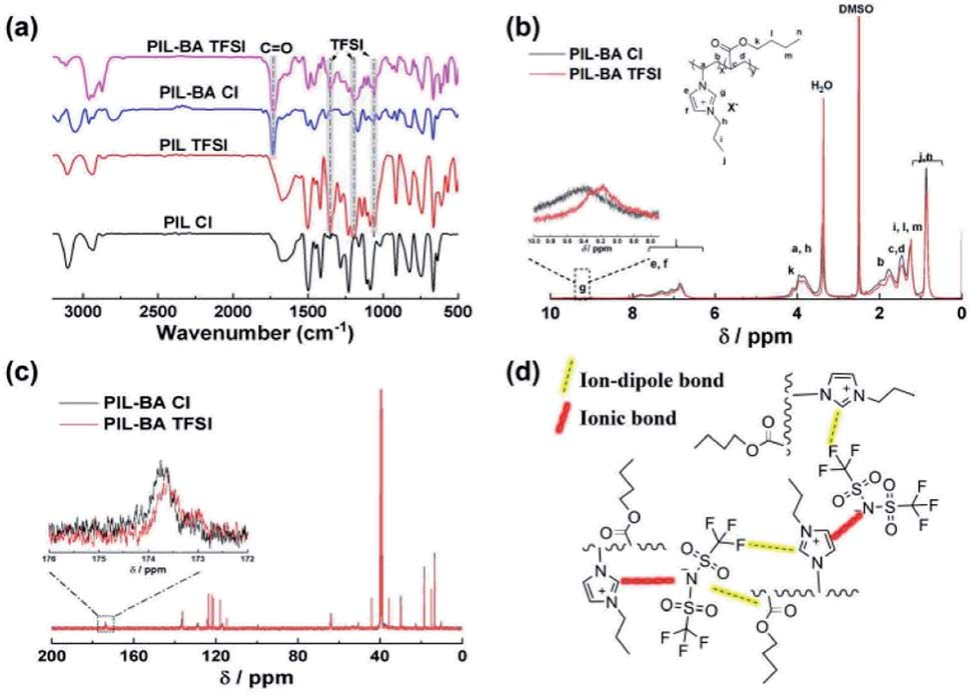
Confirmation of internal interactions in the PIL-BA copolymers. (a) FTIR spectra of the PIL homopolymer and PIL-BA copolymer paired with Cl^−^ or TFSI^−^. (b) and (c) show the ^1^H NMR and ^13^C NMR spectra of the PIL-BA Cl and PIL-BA TFSI, respectively. (d) The illustration of internal interaction in the PIL-BA TFSI system. The yellow dotted line represents ion–dipole bond, the red dotted line represent ionic bond.

Firstly, FTIR, ^1^H NMR and ^13^C NMR spectra were used to gain insight into the interactions between the counter-ions and main chains of PIL-BA copolymer, as shown in Fig. 1. Fig. 1a depicted the FTIR spectra of PIL homopolymers and PIL-BA copolymers containing Cl^−^ or TFSI^−^ as counter-ions. In the spectrum of PIL-BA TFSI, the five characteristic bands attributed to the polymer backbone are observed, assigned to three stretching vibrations of the imidazole ring (3114 cm^−1^, 1622 cm^−1^, and 1275 cm^−1^),^33^ a C=O stretching vibration of ester group (1732 cm^−1^), and a C–O–C stretching vibration of ester group (1111 cm^−1^); the characteristic bands derived from the TFSI^−^ anion, such as an SO_2_ antisymmetric bending (1354 cm^−1^), a CF_3_ antisymmetric bending (1190 cm^−1^) and an antisymmetric stretching vibration of the –SO_2_–N–SO_2_– group (1059 cm^−1^), have also been detected. Compared with the PIL-BA TFSI, the characteristic bands derived from both ester group and imidazole ring of PIL-BA Cl were significantly shifted, seen Table S1,† indicating that there was an obvious change of the interaction between ester group or imidazole ring on polymer backbone and counter-ions in the PIL-BA copolymer systems. In addition, by comparing with the data of PIL TFSI, the characteristic peak of –SO_2_–N–SO_2_– was blue-shifted from 1055 cm^−1^ to 1059 cm^−1^ and the characteristic peak of CF_3_ was blue-shifted from 1198 cm^−1^ to 1190 cm^−1^ in the PIL-BA TFSI, indicating that there was a electrostatic interaction (ion–dipole bond) between the electronegative N or F atoms of TFSI^−^ and ester groups existed in the PIL-BA TFIS. Furthermore, the intensity of interaction between polymer backbone and different counterions (TFSI^−^ or Cl^−^) can be reflected in the corresponding ^1^H NMR (Fig. 1b) and ^13^C NMR spectra (Fig. 1c). The change of chemical shift “g” can be clearly observed in Fig. 1b, which corresponds to the proton in the imidazole ring of the PIL-BA main chain.^32^ Compared with PIL-BA Cl, the proton peak of PIL-BA TFSI shifted to the higher field position, from 9.40 ppm to 9.17 ppm, which indicates that the ionic bond interaction between counterions and the positive imidazole ring was weaker after replacing the Cl^−^ with TFSI^−^. Moreover, ^13^C NMR results (Fig. 1c) show that the characteristic peak of C=O derived from BA shifted from 174.22 ppm to 174.08 ppm, further confirming that adding TFSI^−^ can decrease the interaction intensity between ester group and counterions. Therefore, PIL-BA copolymers with TFSI^−^ counterions have weaker interaction through ionic bonding and ion–dipole bonding, as shown in Fig. 1d. Those results reflected that the larger counter ions (TFSI^−^) associated less and looser, benefiting for the decrease of physical cross-links between polymer chains which would cause a decrease in glass-transition temperature (*T*_g_).^15^

**Fig. 2:**
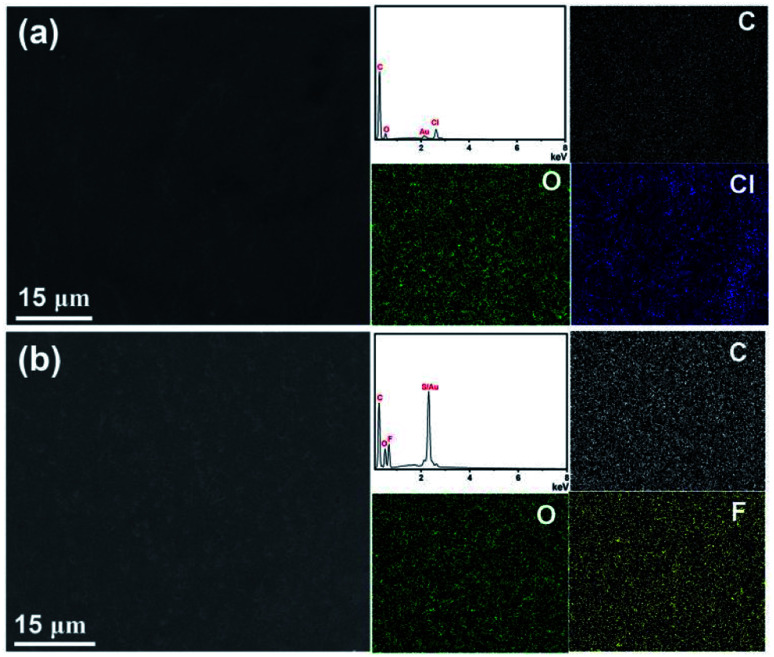
EDS mapping images of PIL-BA Cl (a) and PIL-BA TFSI (b).

Furthermore, the micro-morphology and element analysis of PIL-BA based copolymer films with different counter ions can be confirmed by using the SEM-EDS mapping technique, as shown in Fig. 2. It can be seen clearly that the copolymer films are clean and flat without any holes neither for PIL-BA Cl nor for PIL-BA TFSI. EDS mapping images show that the main elements of PIL-BA Cl film (Fig. 2a) are C, O and Cl and those elements are uniformly distributed on the entire material. The results of element analysis of PIL-BA TFSI (Fig. 2b) are roughly similar to PIL-BA Cl, except that PIL-BA TFSI contains the elements of F and S instead of Cl due to the change of counter ions to TFSI^−^.

**Fig. 3:**
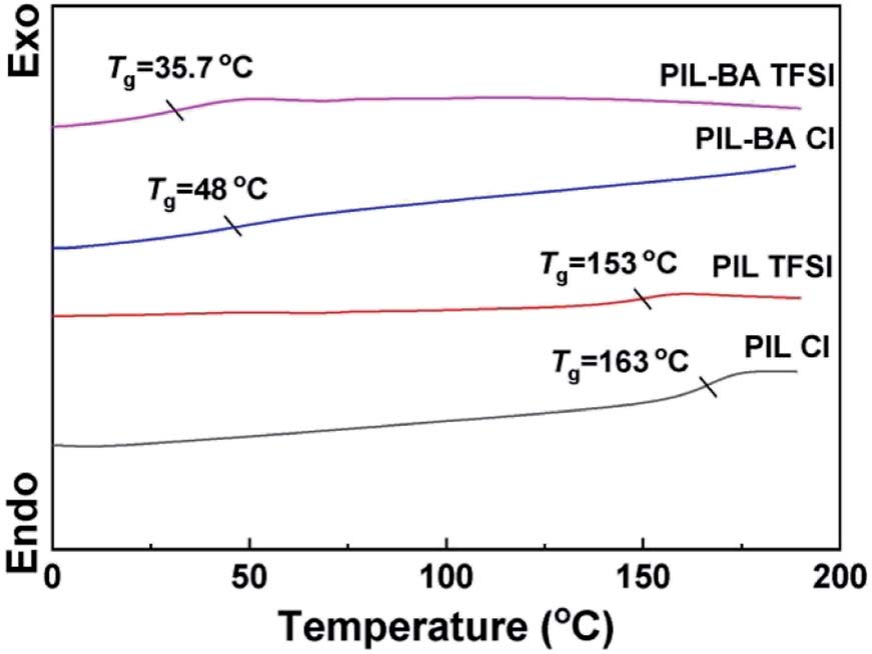
DSC curves of PIL-BA copolymer based electrolytes with different counter-ions.

To investigate the mobility of polymer chains in PIL-BA copolymer based electrolytes, DSC measurements of electrolytes with different counterions were conducted (Fig. 3). PIL Cl homopolymer possessed a high *T*_g_ of 163 °C, thereby its film was very fragile (see Fig. S2†). After exchanging Cl^−^ to TFSI^−^, *T*_g_ value of homopolymer PIL based electrolyte was decreased to 153 °C. Such pronounced decrease in *T*_g_ is mainly caused by the increase of the fractional free volume originated from the larger counter ions (TFSI^−^), which is similar to those reported in literature.^32^ Furthermore, compared with the homopolymer-based electrolytes, PIL-BA copolymer based electrolytes (PIL-BA Cl and PIL-BA TFSI) have very lower *T*_g_ values, and the sample containing TFSI^−^ has the lowest value of *T*_g_ (35.7 °C). It was noting that both of PIL-BA copolymers had a similar powder X-ray diffraction pattern (see Fig. S3†), indicating that the exchanging of anions did not change the amorphous structure of PIL which benefited to the movement of chains. It was demonstrated that both the structural units of BA introduced into PIL main chains and using larger counterions could significantly improve the movement of polymer chains. The synergistic effects can further enhance the motility of chains. Combining the DSC results with structural characterization (Fig. 1), it inferred that the introduction of larger counter ions (TFSI^−^) and flexible segment with BA was beneficial to weaken physical cross-links between molecular chains which caused a decrease in *T*_g_.

**Fig. 4:**
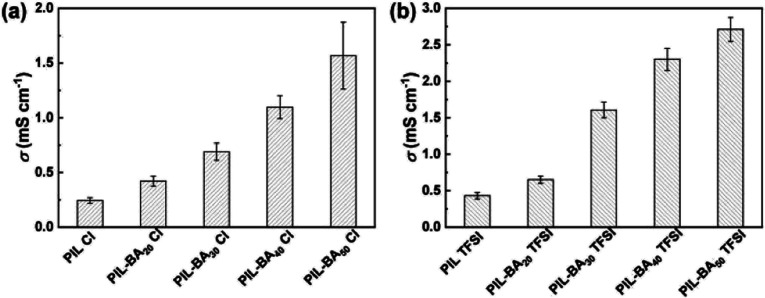
Ionic conductivities (*σ*_s_) of PIL-BA Cl (a) and PIL-BA TFSI (b) copolymer based electrolytes with various feed molar fraction of BA at room temperature.

Before exploring the effect of the different BA content on ion conductive, the molar ratios of ionic liquid monomer and BA incorporated into the copolymers were measured by ^1^H NMR spectra analyses (Fig. S4†). The results are coincident with the initial feed ratios. Moreover, the molecular weights (*M*_w_) and the polydispersity index (PI) of the PIL-BA TFSI based copolymers were tested by gel permeation chromatography (GPC). The GPC analysis shows that the *M*_w_ of the polymers with TFSI^−^ counterions are revealed in the range of 14 000–25 000 and the polydispersity index (PI) of the copolymers are in the range between 1.8 and 2.2 (Table S2†). It is noting that the ion exchange of Cl^−^ with TFSI^−^ does not obviously affect the *M*_w_ of the copolymer at the same molar ratio. The room temperature ionic conductivities (*σ*_s_) of PIL-BA copolymer based electrolytes with different feed molar fractions of BA monomers were studied by EIS measurement (Fig. 4). As shown in Fig. 4a, in the PIL-BA Cl systems, the *σ* significantly increased with the molar fraction of BA and reached 1.57 ± 0.31 mS cm^−1^ at 50 mol% BA (denoted as PIL-BA_50_ Cl), which was 6.5 times higher than that of PIL Cl without BA (0.24 ± 0.03 mS cm^−1^). The room temperature *σ*_s_ of PIL-BA TFSI systems showed same change tendency (see Fig. 4b). It is noted that the *σ* values of samples containing TFSI^−^ were higher than that of the electrolytes containing Cl^−^ at the same molar fraction of BA. As a result, a maximum *σ* value of 2.71 ± 0.17 mS cm^−1^ was found at PIL-BA_50_ TFSI. This phenomenon could be explained by two factors: on the one hand, the larger counterions of TFSI^−^ can weaken the physical cross-link of main chains, leading to the increase of the chain movement which caused to improve the ion transport; on the other hand, the mobility of polymer chains was further improved by introducing BA units into PIL backbones. In addition, the PIL-BA copolymer electrolytes possessed high thermostability that their initial decomposition temperatures were approximately 200 °C (see Fig. S5†). Moreover, the PIL-BA copolymer electrolytes were treated at different pH conditions to examine the chemical stability (see Fig. S6†). Although relative intensity of the groups (such as C=O) of the copolymers were decreased under extreme pH conditions (pH = 1 and pH = 11), no characteristic peaks of the copolymers disappeared, indicating that the PIL-BA copolymers have excellent stability and can adapt to a variety of pH environments.

**Fig. 5:**
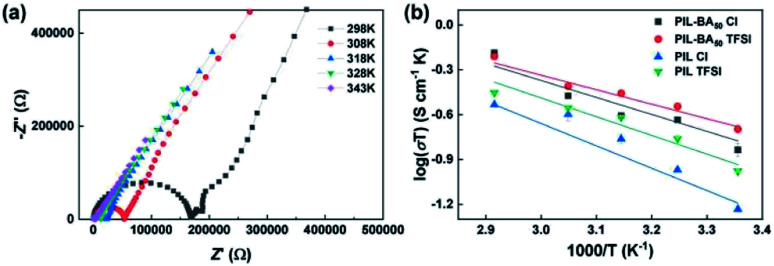
Influence of the temperature on ionic conductivities of PIL-BA copolymer based electrolytes. AC impedance curves (a) and log(*σT*) ∼ 1000/*T* curves (b) at different temperatures.

Furthermore, the ionic conductivity–temperature plots of the homopolymer PIL and copolymer PIL-BA based electrolytes paired with different counterions were conducted in Fig. 5. Fig. 5a shows the temperature-dependent impedance curves of PIL-BA_50_ TFSI electrolyte from 25 °C to 70 °C. The bulk resistance of the electrolyte were decreased markedly with increasing the temperature, thereby the related *σ*_s_ were enhanced. The log(*σT*) ∼ 1000/*T* curves was shown in Fig. 5b and corresponding activation energy (*E*_a_) were summarized in Table S2.† The ionic conductivity–temperature curves of the PIL-BA based electrolytes were reasonably fitted by the Arrhenius behavior. The *E*_a_ values of PIL Cl and PIL TFSI electrolytes are 13.51 kJ mol^−1^ and 11.58 kJ mol^−1^ respectively. The *E*_a_ values of PIL-BA copolymer based electrolytes were obviously lower than that of the homopolymer PIL based electrolytes, and PIL-BA_50_ TFSI electrolyte has the lowest *E*_a_ of 8.68 kJ mol^−1^. The lower *E*_a_ value of PIL-BA_50_ TFSI electrolyte indicates that the movement of ions is less affected by temperature changes, also revealing that the ions of system can be transported easier. Therefore, a fast ion transport channel was formed in the PIL-BA_50_ TFSI system by decreasing *T*_g_ of the polymer.

**Fig. 6:**
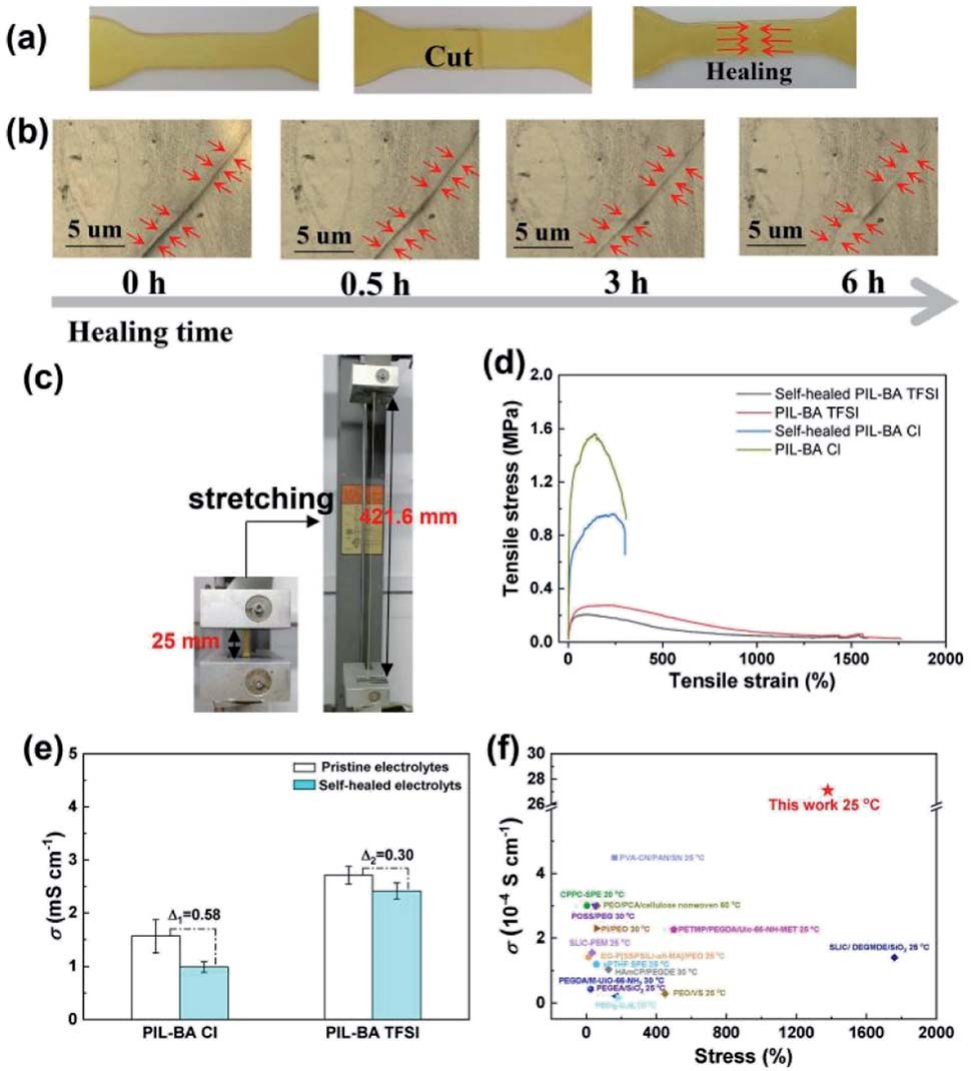
Photos (a) and optical microscope images (b) of PIL-BA_50_ TFSI electrolytes that were cut into two pieces and then healed at 40 °C for 6 h; (c) photographs showing the high-stretching ability of the healing sample; (d) stress–strain curves of the intact and self-healed PIL-BA_50_ TFSI electrolytes comparing with PIL-BA_50_ Cl; (e) the ionic conductivities of the pristine and self-healed PIL-BA based electrolyte; (f) comparison of the toughness and ionic conductivity of PIL-BA TFSI to other reported polymer electrolytes.

Because of the superior flexibility of the polymer chains and the weak electrostatic interactions between TFSI^−^ counterions and polymer backbones in the PIL-BA_50_ TFSI system, the PIL-BA_50_ TFSI membranes exhibit self-healing ability. As shown in Fig. 6a, the cut PIL-BA_50_ TFSI membrane can be self-healed together at 40 °C for 6 h. The optical microscope images in Fig. 6b reveal that the scar is finely healed and gradually disappeared after 6 h at 40 °C. As further evidenced by the tensile tests, the healing sample could withstand stretching to a large extension (from 25 mm to 421.6 mm), shown in Fig. 6c. In addition, the PIL-BA polymer electrolyte containing Cl^−^ counterion was also able to self-heal at 40 °C due to the ionic aggregation behavior.^14,23^Fig. 6d compared the stress–strain curves of the cut PIL-BA_50_ based samples with different counterions (Cl^−^ and TFSI^−^). Those cut polymer electrolytes were self-healed at 40 °C for 6 h. The tensile strength of the cut-healed PIL-BA_50_ TFSI and PIL-BA_50_ Cl electrolytes can reach ∼75% and ∼61% of the original values for the intact samples. This observation revealed that the counter ion of TFSI^−^ increased significantly the healing performance because that TFSI^−^ ions acting as the plasticizers resulted in a remarkable enhance the mobility of polymer chains, which was in accordance with the DSC results. Moreover, the strain value at break of the PIL-BA_50_ TFSI was much higher than that of the PIL-BA_50_ Cl, reaching 1762%. Dynamic mechanical analysis (DMA) measurement reveals that the loss modulus (*G*′′) and the storage modulus (*G*′) of the PIL-BA_50_ TFSI polymer electrolyte were lower than that of the PIL-BA_50_ Cl across the frequency range of 0.1 to 10 Hz (Fig. S7†) due to the decrease in *T*_g_, indicating that the PIL-BA_50_ TFSI polymer electrolyte benefits for the self-healing. Therefore, the obtained PIL-BA_50_ TFSI polymer electrolyte exhibited higher recovery of the moduli than that of the PIL-BA_50_ Cl upon cutting and self-healing. More importantly, the PIL-BA_50_ TFSI polymer electrolyte not only shows better mechanical healed performance as compared to the PIL-BA_50_ Cl polymer electrolyte, but also revealed higher healed performance of ionic conductivity than the latter (Fig. 6e). It is also worthwhile noting that PIL-BA_50_ TFSI has much higher strain capacity and ionic conductivity than most reported polymer electrolytes (Fig. 6f),^34–47^ indicating promising application prospects in all-solid-state electrochemical devices to ensure high security during the long-term cycles.

## Experimental

### Synthesis of poly(ionic liquid) (PIL) copolymer with different counterions

Poly(ionic liquid)-*co*-butyl acrylate chloride (denoted as PIL-BA Cl) was first synthesized *via* radical polymerization of *N*-vinylimidazole-based ionic liquid (VIL) and butyl acrylate (BA) monomers using 2,2-azobis(2-methylpropionitrile) (AIBN, Aladdin agent) as the initiator. The molar ratio of VIL/BA was varied from 5 : 5, 6 : 4, 7 : 3, and 8 : 2. Firstly, the VIL was synthesized by the previously reported methods.^20^ Briefly, distilled methylbenzene (8 g), chloropropane (3.14 g, AR, Aladdin agent), and *N*-vinylimidazole (1.88 g, AR, Aladdin agent) were mixed in a 50 mL round-bottomed flask under nitrogen atmosphere, followed by stirring and refluxing the mixture at 50 °C for a period of 12 h. After that, based on the different molar ratios of VIL and BA, a certain weight of BA was added for stirring 10 min at 50 °C. Finally, the initiator of AIBN dissolved into 5 mL methylbenzene was dropwise added into the reaction mixture under vigorous stirring at 65 °C for 48 h. The initiator accounts for 2% of the total monomers. After the polymerization process, the resulting precipitate was filtrated, and repeatedly washed for several times with anhydrous ether, and further purified *via* the Soxhlet extraction and then dried in a vacuum oven at 50 °C. As a contrast, the PIL homopolymers without BA were also synthesized.

Poly(ionic liquid)-*co*-butyl acrylate bis(trifluoromethanesulfonyl)imide (denoted as PIL-BA TFSI) was prepared *via* ion exchange metathesis of the above-mentioned PIL-BA Cl copolymers. The PIL-BA copolymer paired Cl^−^ (2.0 g) was dissolved in 10 mL deionized water under vigorous stirring to obtain a precursor aqueous solution. Then 7.5 mL aqueous solution of LiTFSI (0.4 M) was slowly added to the precursor aqueous solution dropwise under vigorous stirring for 24 h to exchange the counteranions of the PIL-BA copolymers into TFSI^−^. The resultant precipitate was filtered and thoroughly washed with deionized water to remove the excessive LiTFSI and then dried in a vacuum oven at 50 °C.

The as-prepared PIL copolymers are denoted as PIL-BA_*a*_ X, where a represents the feed molar fraction of BA monomers in the copolymer, and X represents the counteranions (X: Cl^−^, or TFSI^−^).

### Preparation of PIL-BA copolymer electrolyte membranes

Electrolyte membranes of PIL-BA copolymer with different counter-ions were fabricated by the solvent casting method. The as-synthesized PIL-BA copolymers (1.00 g) were first dissolved into 10 mL absolute ethyl alcohol and stirred for at least 24 h at room to obtain a precursor aqueous solution (see Fig. S1†). At that same time, the ionic liquid mixture electrolyte consisted of 15 wt% lithium salt (Li-IL) was prepared by dissolving lithium bis(trifluoromethylsulfonyl)imide (99.95%, Sigma-Aldrich) in 1-ethyl-3-methylimidazolium bis(trifluoromethylsulfonyl)imid (99%, Innochem). Then a certain amount of Li-IL was added to the precursor solution dropwise under vigorous stirring for 24 h and subsequently casting onto Teflon substrates (*ca.* 50 mm (*L*) × 25 mm (*D*) × 3 mm (*T*)). The mass fraction of the Li-IL in the copolymer electrolytes were 30 wt%. The cast films were partially allowed to evaporate under ambient conditions in the box filled with argon for 24 h. Finally, the polymer electrolyte membranes were entirely dried under vacuum at 50 °C for 48 h. For testing the ionic conductivity, the membranes were cut into circular samples with a diameter of 16 mm.

### Characterizations

Fourier-transform infrared (FT-IR) spectra were conducted on a Bruker Tensor 27 spectrometer in attenuated total reflectance (ATR) mode. For testing the chemical stability of the prepared copolymer electrolytes, the samples were added into different pH values of the aqueous solutions and stirred for 3 days at room temperature. After that, the treated samples were dried and tested by the FT-IR. The NMR spectra of monomers and polymers were recorded on a Bruker-400 MHz spectrometer (399.65 MHz for ^1^H NMR and 125 MHz for ^13^C NMR, respectively). The glass transition temperatures (*T*_g_s) of the samples was recorded and analyzed on a differential scanning calorimeter (DSC, TA/Q10). Thermogravimetric analysis (TGA) was performed using a TA Instruments Q50 system from 40 to 600 °C at the heating rate of 10 °C min^−1^ under nitrogen atmosphere. The molecular weight (*M*_w_) and polydispersity index (PI) of the prepared copolymers were measured by gel permeation chromatography (GPC, Agilent PL-GPC50) with the DMF containing 50 mM LiBr as the eluent. The stress–strain test of the electrolyte samples was carried out on a CMT6103 tensile testing machine with a tensile speed of 10 mm min^−1^ at 25 °C. Dynamic mechanical analysis (DMA) measurements were conducted on a TA Instruments DMA 850 with frequency sweeps at 0.1–10 Hz at 25 °C. Healing experiments were tested by gently bringing the cut pieces back into contact at 40 °C. Scanning electron microscopy (SEM) images of the samples were obtained using a Hitachi S-4800 field emission electron microscope equipped with an energy dispersive X-ray fluorescence spectrometer for element analysis.

The ionic conductivities (*σ*_s_) of the electrolytes at different temperatures were calculated using a CHI 660E electrochemical workstation (CH Instruments Inc.) in a coin-cell composed of the electrolyte membranes, two stainless steel as electrodes, using the AC impedance method in the frequency range from 1 MHz to 1 Hz with an amplitude of 5 mV at an open-circuit potential. The values of *σ* were calculated using the following equation: *σ* = *L*/*R*_S_ × *A*, where *L*, *R*_S_ and *A* were the spacing between two electrodes, the bulk resistance, and the area of the electrode–electrolyte contact, respectively.

## Conclusions

In summary, a high ionic conductivity, flexible and self-healing polymer electrolyte was fabricated by copolymerizing imidazolium monomers with butyl acrylate followed with TFSI^−^ as counteranions. A series of structure characterization proves that an interaction exists between the counter ions and the polymer backbones, which is directly affected by the type of counterions. The introduction of BA units and TFSI^−^ counteranions gave rise to a low glass transition temperature and free mobility of poly(ionic liquid) (PIL) chains, thus significantly improved the ion conductive (2.71 ± 0.17 mS cm^−1^) with high toughness (tensile strain of 1762%) and good self-healable capability. The PIL-BA copolymer based electrolyte developed in this study may give a new idea to design the molecular structures of polymer electrolytes for secure and stable flexible electronics.

## Author contributions

Fu Jie Yang: conceptualization, writing – original draft, writing – reviewing & editing. Qing Feng Liu: formal analysis, visualization, investigation. Xiao Bing Wu: validation, data curation. Yu Yi He: investigation. Xu Gang Shu: project administration, supervision. Jin Huang: writing – reviewing, resources, funding acquisition.

## Conflicts of interest

There are no conflicts to declare.

## Supplementary Material

RA-011-D1RA04553A-s001
