# Supplementary material for: High ionic conduction, toughness and self-healing poly(ionic liquid)-based electrolytes enabled by synergy between flexible units and counteranions
Source: RSC Adv. 2021 Nov 3;11(56):35687–94. doi: 10.1039/d1ra04553a (PMC9043274; doi:10.1039/d1ra04553a)
Supplement: RA-011-D1RA04553A-s001 [file RA-011-D1RA04553A-s001.pdf]

Electronic Supplementary Material (ESI) for RSC Advances.
This journal is © The Royal Society of Chemistry 2021

Supporting Information for

High Ionic Conduction, Toughness and Self-healing Poly(Ionic Liquid)-based Electrolytes Enabled by Synergy between Flexible Unit and Counteranion

Fu Jie Yang‡*[a], Qing Feng Liu‡[a], Xiao Bing Wu[a], Yu Yi He[a], Xu Gang Shu[a] and Jin Huang*[b]

[a]College Chemistry and Chemical Engineering, Zhongkai University of Agriculture and Engineering, Guangzhou 510275, P. R. China

[b]College of Pharmacy, Guangxi Medical University, Nanning 530021, P. R. China.

*E-mail: yangfujie580@163.com; huangjin@mailbox.gxnu.edu.cn

‡ Fu Jie Yang and Qing Feng Liu contributed equally to this work.

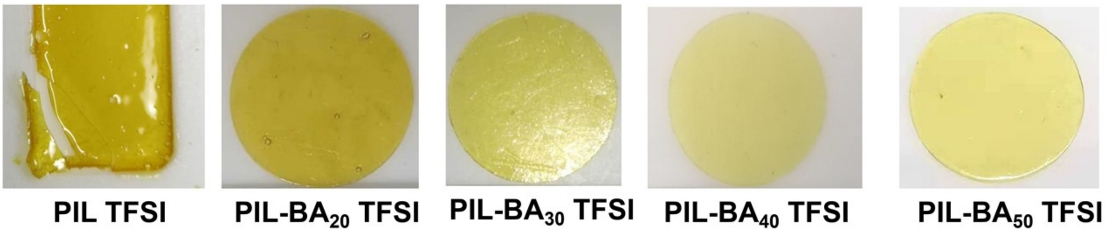

Figure S1. PIL-BA TFSI electrolyte films with different content of BA.

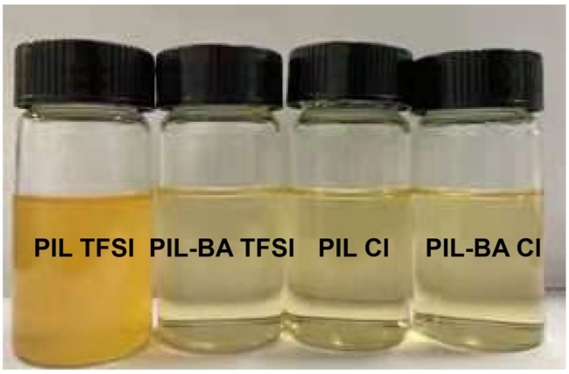

Figure S2. Photographs of PIL and PIL-BA ethanol solutions containing different counter- ions (Cl^- and $TFSI^-$).

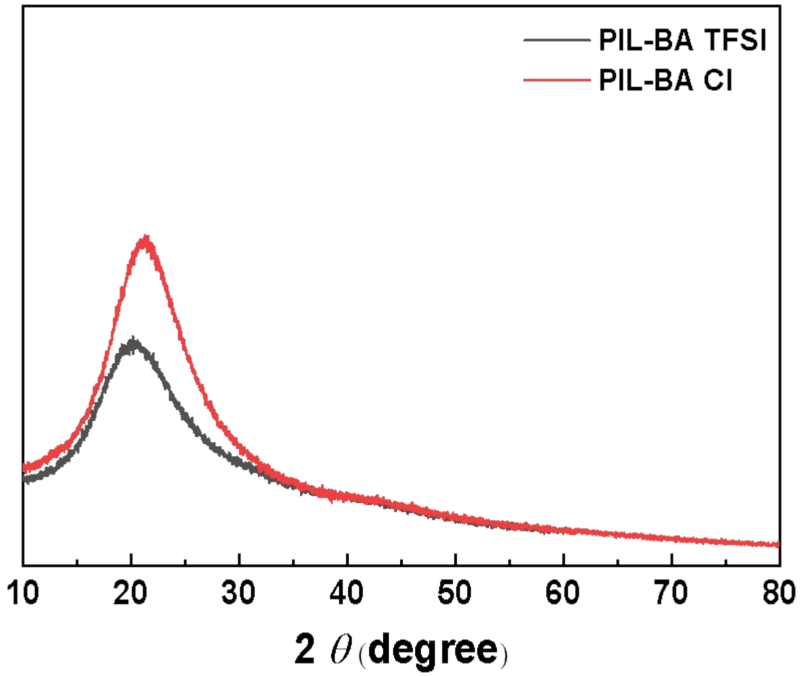

Figure S3. XRD patterns of PIL-BA copolymer paired with Cl^- and $TFSI^-$.

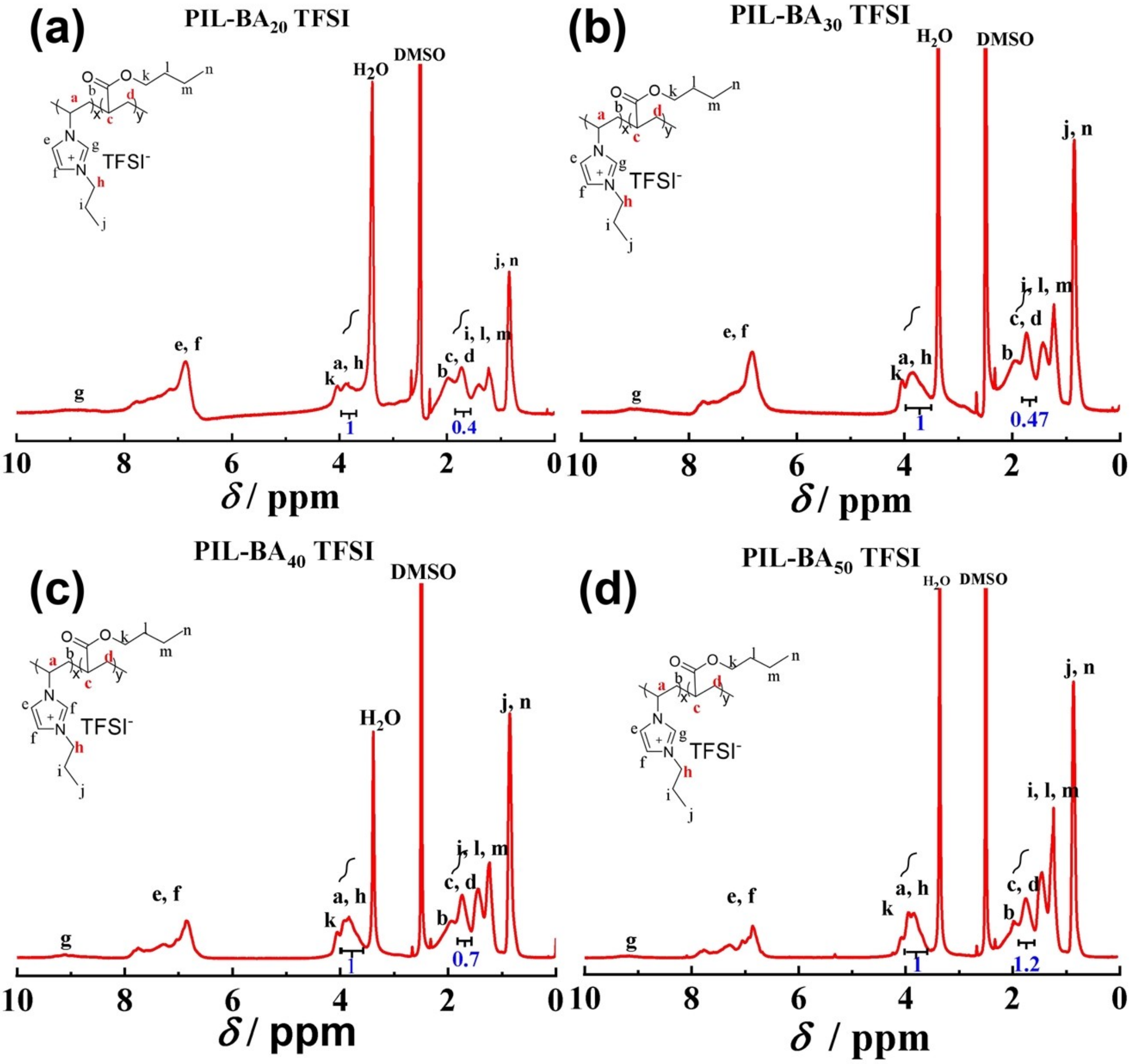

Figure S4. ^{1}H NMR spectrum of PIL-BA TFSI with different molar ratios of ionic liquid monomer and butyl acrylate.

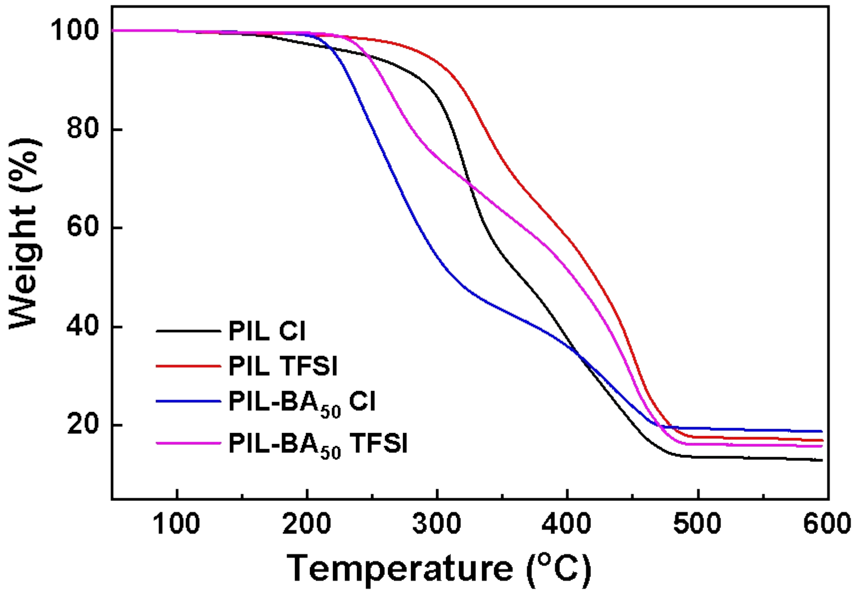

Figure S5. TG curves of various PIL based electrolytes.

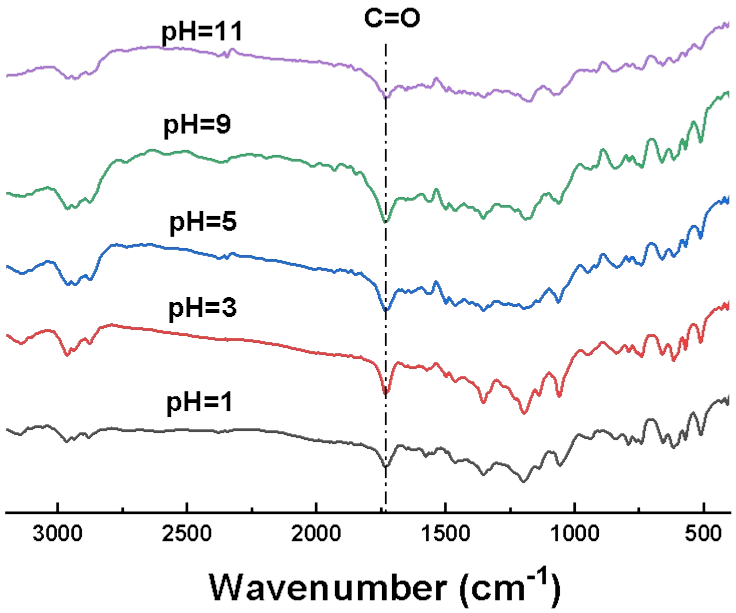

Figure S6. FTIR spectra of PIL-BA Cl copolymer electrolytes which were treated under different pH conditions.

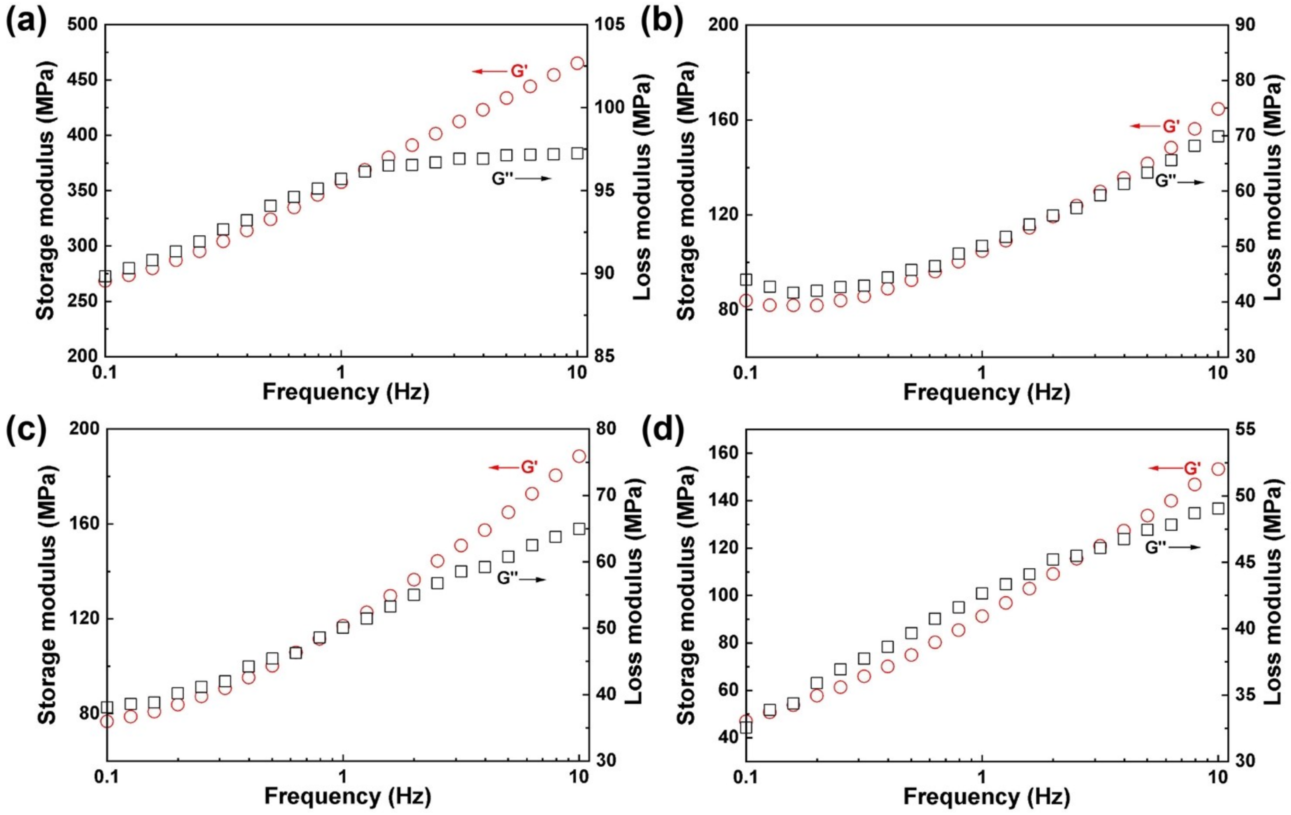

Figure S7. Storage mudulues (G') and loss mudulus (G'') of the PIL-BA based polymer electrolyte films: (a) and (b) represent PIL-BA Cl before and after self-healing; (c) and (d) represent PIL-BA TFSI before and after self-healing.

Table S1. Position and assignment of the important FTIR signals.

**Samples**	**Imidazole ring**			**Ester group**	
	ν(HC=CH) / cm^{-1}	ν(C=N) / cm^{-1}	ν(C-N) / cm^{-1}	ν(C=O) / cm^{-1}	ν(C-O-C) / cm^{-1}
PIL-BA TFSI	3114	1622	1275	1732	1111
PIL-BA Cl	3167	1626	1265	1734	1113

Table S2 Molecular characteristics of PIL-BA TFSI copolymers

**Samples**	M_w	M_w/M_n
**PIL-BA$_{20}$ TFSI**	**14000**	**1.8**
**PIL-BA$_{30}$ TFSI**	**19000**	**1.9**
**PIL-BA$_{40}$ TFSI**	**23000**	**1.9**
**PIL-BA$_{50}$ TFSI**	**25000**	**2.2**
**PIL-BA$_{50}$ Cl**	**23000**	**2.2**

Table S3 The corresponding activation energy (E_a) of ice-templated GO / PIL composite electrolytes.

Samples	Activation energy (E_a, kJ mol^{-1})
PIL Cl	13.51
PIL TFSI	11.58
PIL-BA_{50} Cl	9.65
PIL-BA_{50} TFSI	8.68
